# *I need it. Without it, I wouldn't have gone, and I do want to go*: patient perspectives on the acceptability and appropriateness of two patient-centered interventions to improve adherence to annual lung cancer screening

**DOI:** 10.3389/frhs.2026.1810956

**Published:** 2026-09-23

**Authors:** Lorella Palazzo, Casey Luce, Anjali Vasavada, Kayne Mettert, Hongyuan Gao, James D. Ralston, Nadejda Bezman, Beverly B. Green, Kristine Rogers, Melissa L. Anderson, Yu-Ru Su, Matthew Triplette, Lisa Carter-Bawa, Karen J. Wernli

**Affiliations:** 1 Kaiser Permanente Washington Health Research Institute, Seattle, WA, United States; 2 University of Washington, Seattle, WA, United States; 3 Department of Health Systems and Population Health, University of Washington, Seattle, WA, United States; 4 Department of Health Systems Science, Kaiser Permanente Bernard J. Tyson School of Medicine, Pasadena, CA, United States; 5 Fred Hutchinson Cancer Center, Seattle, WA, United States; 6 Center for Discovery and Innovation at Hackensack Meridian Health, Nutley, NJ, United States; 7 Georgetown Lombardi Comprehensive Cancer Center, Washington, DC, United States

**Keywords:** acceptability, appropriateness, cancer screening, health communication, implementation, lung neoplasm, patient, pragmatic clinical trial

## Abstract

**Introduction:**

Low adherence to annual lung cancer screening (LCS) has tempered mortality benefits in clinical practice. Herein, we assessed the acceptability and appropriateness of two patient-centered interventions addressing adherence barriers to annual LCS.

**Methods:**

Participants were Kaiser Permanente Washington members, aged 50–78 years, who participated in a pragmatic randomized trial (“The Larch Trial”). The trial tested: (1) health communication including a video to improve patient knowledge of timeliness to return; and (2) outreach reminders to patients to schedule annual screenings after their primary care provider (PCP) ordered a scan without an appointment. Participants, randomly sampled on sex, race/ethnicity, tobacco status, and trial arm, completed semistructured, virtual interviews that lasted for 60 min. The interviews explored intervention acceptability and appropriateness, and barriers and facilitators to both. Transcripts were analyzed using a rapid group analysis process informed by the Consolidated Framework for Implementation Research. Identified themes were second-coded for acceptability and appropriateness in accordance with the Implementation Outcomes Framework.

**Results:**

A total of 32 participants were interviewed; 66% were non-Hispanic white, 53% male, and 53% currently use tobacco. The participants found the interventions acceptable and appropriate with variations by LCS history and information needs. Some participants found the video helpful and comforting, especially first-time screeners. Other participants, particularly those engaged in annual LCS, found that the video lacked new information. The participants strongly endorsed the reminder intervention for encouraging LCS and clarifying the timing of repeat screening. Most participants endorsed the annual screening scan ordered without a PCP visit. The participants preferred the multimodal delivery of reminders for reinforcement. Facilitators of acceptability and appropriateness included video design and perceived educational value, and an electronic health record ecosystem supporting communication aligned with routine practice. Barriers included content perceived as not novel, lack of information to address specific needs, and excessive reminders.

**Discussion:**

Video-based content in LCS is generally acceptable and appropriate to patients if tailored by LCS history. Patient outreach reminders are acceptable and appropriate when mirroring other healthcare system communications. Participants already engaged in LCS did not feel they needed to see their PCP one year later for repeat screening (within 1 year). Future studies should test interventions in other populations and healthcare systems using different communication strategies.

**Clinical trial registration:**

clinicaltrials.gov, identifier #NCT05747443.

## Introduction

Lung cancer is the leading cause of cancer death in the US (1, 2). For more than10 years, the US Preventive Services Task Force has recommended annual screening with low-dose computed tomography (LDCT) for those aged 50–80 years with a significant tobacco history (3). Despite its public health promise, uptake of lung cancer screening (LCS) remains low (5%–20%) (4–6), and adherence to annual LCS varies from 22% to 60% (7, 8). Patient barriers to LCS adherence include unawareness of the need for repeat LCS, experience of tobacco-related stigma, and fear of lung cancer diagnosis (9, 10). Furthermore, at the clinician- and system level, direct patient outreach when patients are due for LCS is often lacking (11). To date, few clinical trials have tested interventions to directly address these known barriers.

Evidence from other cancer screening domains suggests that multimedia interventions (e.g., informational videos) and patient-directed reminders can improve repeat screening (12). Video-based health communication interventions have several advantages, including standardized delivery and the potential to manifest empathy, and are effective in improving short-term knowledge (13). Furthermore, patient-directed reminders tailored to the time when they are due can be delivered multimodally (e.g., mail and telephone) and through standardized workflows. These types of interventions can be easily and efficiently delivered as digital tools through the electronic health record (EHR) patient portal. However, limited empirical evidence exists on the effectiveness of LCS digital health tools to improve knowledge, increase uptake, or improve adherence (14).

Herein, we conducted a pragmatic clinical trial (Larch Trial) to test two patient-centered interventions to improve adherence to annual LCS (main outcome) (15, 16). Trial interventions addressed patient knowledge gaps through a health communication intervention centered on a video and through reminders directed toward patients when they were due for LCS (10). However, to date, we have limited evidence on how to design and deploy digital communication in LCS that reflects patient preferences, needs, and priorities, particularly focused on adherence to annual screening. Collecting patients’ views through qualitative methods can help address this broad research question: What accounts for the successful implementation of patient-centered video- and reminder-based digital health interventions to improve completion of annual LCS?

The objective of this interview study was to explore the acceptability and appropriateness of trial interventions to patients and examine the factors that influence patient-centered implementation to inform LCS programs in the US. We used the Implementation Outcomes Framework (IOF) (17, 18) and the Consolidated Framework for Implementation Research (CFIR) (19, 20), which are foundational frameworks, and which offer theoretical grounding and constructs to examine trial implementation systematically from a patient’s perspective.

## Materials and methods

### Study design and data sources

We conducted interviews with participants in the Larch Trial using a semistructured interview guide (Supplementary material 1). Interviews lasted up to 60 min. Although the CFIR and IOF were used to structure the overall study, elements of descriptive phenomenology informed the interview approach to ensure attention to patient experience (21, 22). We followed the guidelines of the Consolidated Criteria for Reporting Qualitative Research to present our findings (23) (Supplementary material 2). This study was reviewed and approved by the Kaiser Permanente Interregional IRB (IRB #1914783).

### Theoretical frameworks

The CFIR and IOF provided a conceptual foundation and structured data analysis and interpretation. As a determinants framework with five domains (i.e., innovation, outer setting, inner setting, individuals, and process) and multiple constructs, the CFIR supported a systematic account of contextual influences on patients’ experiences with LCS and perceptions of trial interventions (24). Using recipient-centered constructs from the updated CFIR enabled a comprehensive understanding of patient needs and preferences for interventions supporting LCS adherence, as well as multilevel implementation barriers and facilitators.

The IOF operationalizes implementation outcomes at the individual and organizational level. In this study, IOF constructs guided our assessment of patient-level acceptability and appropriateness, defined as perceptions of whether each intervention was agreeable and aligned with patients’ needs.

### Larch Trial study population and interventions

Details of the trial and interventions are published elsewhere (15, 25). Briefly, 1,837 members of Kaiser Permanente Washington (KPWA, an integrated delivery system in the Pacific Northwest) aged 50–78 years who completed LCS with negative findings were randomized to one of four groups, to evaluate two interventions using a 2 × 2 factorial study design. The study interventions consisted of a health communication intervention centered on Patient Voices Video (https://kpwashingtonresearch.org/kplung), coupled with printed messaging, and a Stepped Reminders intervention. Participants were randomized to receive (a) neither intervention; (b) health communication intervention only; (c) Stepped Reminders only; or (d) both interventions.

The video intervention was developed in collaboration with professional designers and conveyed key messages relevant to LCS and patients’ informational and social needs that surfaced in previous work (10). Printed communication contained the same messages (Figure 1). Video design included accessible and inclusive language (e.g., subtitles in Spanish) and images representing diverse patients. Length, pace, and overall tone were chosen to relay calmness and promote broad appeal.

**Figure 1:**
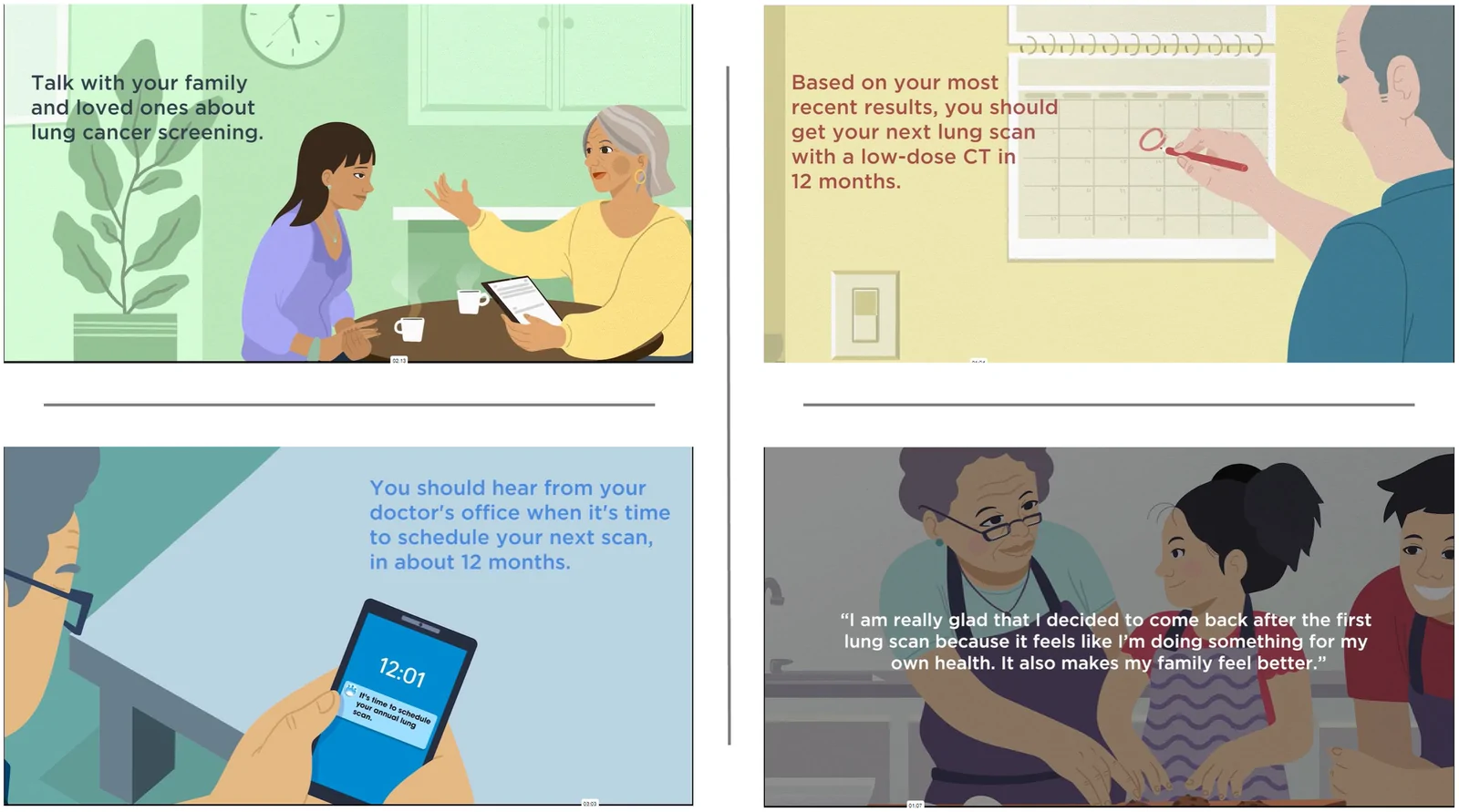
Key messages conveyed in the Patient Voices Video.

This health communication intervention was designed to address patient educational needs regarding LCS, beyond what was communicated through shared decision-making (SDM). Three weeks after a negative LDCT scan result, which qualified patients for trial entry, the video was delivered. Key messages of the intervention were to normalize routine screening, remind patients when was their next due, and encourage social support. The Stepped Reminders intervention addressed patients’ need to be reminded to schedule a repeat LDCT about 4 weeks before they were due for annual screening (Figure 2). In the intervention, the study's LCS coordinator pended an order for LCS LDCT to the patient's primary care provider (PCP) to sign and, when signed, initiated outreach to patients to schedule LDCT with the Radiology department (15). Participants were tracked up to 15 months after the index LDCT (which qualified them for the trial) for receipt of repeat LCS LDCT (main trial outcome).

**Figure 2:**
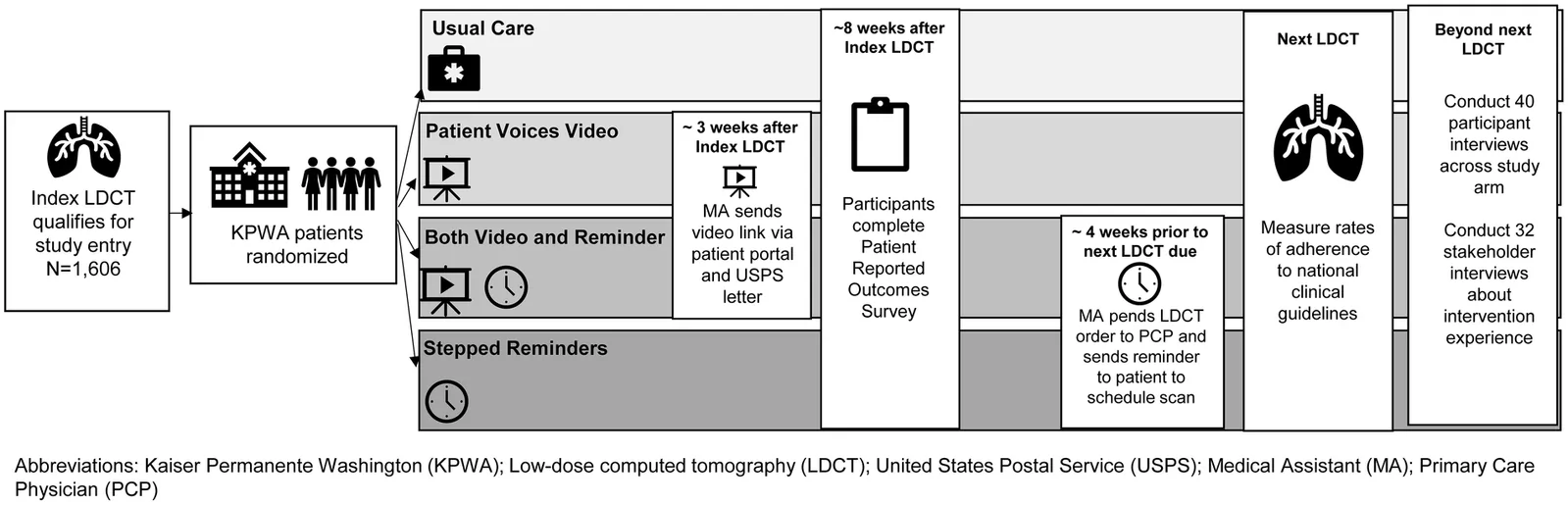
Larch Trial study flow with timing of intervention delivery. Reproduced with permission from “Study flow diagram of randomization, intervention, and outcome assessment of study trial 2022–2024” by Casey Luce, Lorella Palazzo, Melissa L. Anderson, Lisa Carter-Bawa, Hongyuan Gao, Beverly B. Green, James D. Ralston, Kristine Rogers, Yu-Ru Su, Leah Tuzzio, Matthew Triplette and Karen J. Wernli.

To ensure alignment with KPWA communication ecosystem and norms, interventions were delivered primarily through the EHR patient portal and augmented by mailed letters. Stepped Reminders were further augmented by telephone communications.

### Interviews

#### Interview recruitment

Participants were eligible for interview when the 15-month follow-up was complete. A planned *N* = 32 sample size (8 per trial arm) was considered feasible and adequate to address the research questions (26, 27).

As participants became eligible, the trial programmer identified the participant sample by random selection based on trial arm, sex (male/female), race and ethnicity (non-Hispanic white/person of color), and tobacco status (current use/former use of tobacco). Non-white participants were oversampled. Random sampling within categories was used to reduce the influence of selection bias.

Potential participants were mailed a recruitment letter and interview information sheet to invite them to complete the interviews. Trained staff followed up with potential participants twice by telephone to schedule interviews. Participants received $60 incentives for interview completion. Multimodal outreach, accessible information materials, scheduling responsive to potential participants’ needs, and offer of an incentive were used to limit the chances of recruitment non-response (28).

#### Interview procedures

We interviewed participants who were representative of all trial arms. Before beginning the interviews, facilitators described the research study and interview purpose and procedures. The participants provided oral consent for the interviews. There was no prior relationship between researchers and interview participants. We developed and iterated a semistructured interview guide focused on assessing acceptability and appropriateness of trial interventions, with additional questions to understand implementation context, including participants’ experiences with LCS, how such screening compared with other preventive care (e.g., other cancer screening), participants’ relationship and communication with their PCP, and barriers and facilitators to LCS adherence. The guide was piloted with members of the Larch Trial Patient Advisory Board (PAB), a 3-member board of KPWA patient partners eligible for LCS, and feedback was incorporated. Because the participants were representative of all arms and may or may not have received and/or be able to recall trial interventions, interviewers showed all participants slides displaying vignettes and key messages from the video (Figure 1) as well as the flow and timing of delivery (Figure 2) and asked for contemporary perspectives rather than recall of receipt of interventions.

The interviews lasted up to 60 min, were conducted through Microsoft Teams, audio-recorded with permission, and transcribed.

After completing the interviews, the interviewers generated summaries through a prestructured interview debrief form to synthesize key points and document any emerging insights for discussion with the research team.

CL and AV facilitated the interviews, with no other team member present. They and LP led the data analysis. All had graduate-level training and experience in qualitative methods and were employed by KPWA during the study. The other team members had expertise in epidemiology, clinical psychology, implementation science, informatics, biostatistics, and public health. Four were clinicians at KP or other health systems. The full research team gave feedback on the analytic plan and interview guide and supported data analysis and interpretation.

### Data analysis

Data analysis proceeded in three phases: In Phase 1, the research team developed a code list comprising CFIR-informed and inductive codes. Phase 2 consisted of a rapid group analysis process to analyze transcripts across all interview participants and identify crosscutting themes (29). In Phase 3, identified deductive and inductive themes and related quotes were second-coded for acceptability and appropriateness to participants (30–32) (Figure 3).

**Figure 3:**
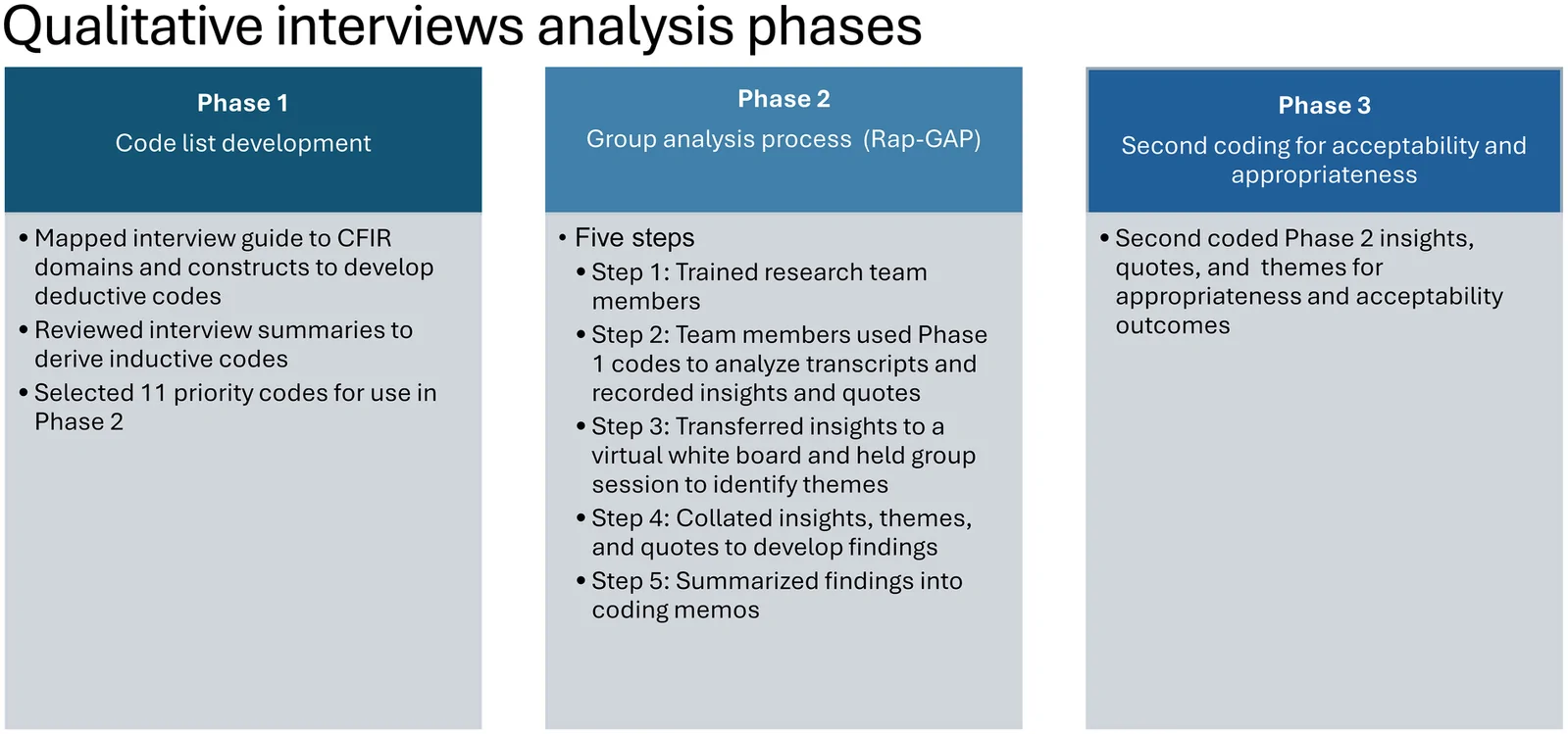
Phases and key steps in patient interview analysis.

We monitored thematic saturation throughout the data collection process by reviewing interview summaries and discussing emerging findings in team debriefs. This process helped determine whether the data gathered adequately addressed the primary research question. If saturation was reached, then no additional recruitment was deemed necessary beyond the planned sample.

In Phase 2, ongoing group-based interpretation and discussion enabled assessing whether new themes continued to emerge. Analytic reliability was pursued through specific steps across Phases 1–3 as described in the sections below.

#### Phase 1: code list development

Four researchers (LP, CL, AV, and KM) collaboratively developed the code list for analysis by mapping CFIR constructs to interview questions to develop deductive codes and then adding inductive codes derived from interview debrief summaries. First, the researchers identified CFIR domains applicable to the study, mapped interview guide questions to constructs within the domains, and renamed the constructs to mirror interview topics (Table 1). Second, the researchers reviewed interview debrief notes and summaries to identify inductive codes. The collaborative, discussion-and consensus-driven approach promoted the development of valid and reliable codes. The resulting deductive and inductive code list was iteratively refined and finalized.

**Table 1:** Updated CFIR constructs mapped to interview guide questions.

CFIR relevant domain and construct	Adapted CFIR construct	Adapted CFIR construct definition	Sample patient interview questions mapped to CFIR constructs
Innovation—C. Innovation relative advantage	Relative advantage of Larch Trial interventions	One of the two Larch Trial interventions (Patient Voices Video or Stepped Reminders) is better or worse than the other. Or, one or both interventions are better or worse than current LCS care	How does receiving reminders for lung cancer screening compare with receiving reminders from KPWA for other tests (e.g., routine blood work and other preventive screenings)?
Innovation—G. Innovation design	Video content and design	The Patient Voices Video is well designed and packaged, including how content is presented, as reflected in the patient's experience with or reaction to Patient Voices Video content and design	What suggestions do you have to improve the experience of receiving and watching Patient Voices Video? […]
Innovation—G. Innovation design	Reminders’ structure and design	The Stepped Reminders intervention is well designed and packaged, including how the steps are assembled and the reminders delivered	What is your reaction to the Stepped Reminders program I just described? What did you find appealing or helpful? What would you change?
Inner Setting—D. Culture—2. Recipient-centeredness	Patient experience with KPWA PCP	Patient experience regarding the KPWA norms of caring, supporting, and addressing the needs and welfare of patients as reflected in the relationship and interactions with the PCP	Tell me about your experience and your rapport with your PCP. Do you feel you can trust this provider?
Individuals (characteristics subdomain)—A. Need	Patient needs regarding LCS	Patient needs and care gaps with the current lung cancer screening process to be addressed by the Larch Trial interventions	What do you think is important for your PCP to know about you when you are thinking about lung cancer screening?
Individuals (characteristics subdomain)—C. Motivation	Patient motivation	Patient commitment to lung cancer screening	What would motivate you to continue lung cancer screening?
Implementation process—B. Assessing needs—2. Innovation recipients	Larch Trial interventions meeting patients’ needs	Collect patient priorities, preferences, and needs to guide the implementation and delivery of Larch Trial interventions	Would you want to talk to your PCP about watching Patient Voices Video and initiate a conversation with them about lung cancer screening? Why or why not?
Implementation process—C. Assessing context	Patient experience with the current LCS process	Collect information on patient experience with barriers and facilitators to lung cancer screening adherence to guide the implementation and delivery of Larch Trial interventions	What (if anything) gets in the way of returning for your next screening scan when you are due? […]

#### Phase 2: rapid group analysis process

Phase 2 of the analysis centered on Rap-GAP, a group-based, rapid analysis process in which team members and other participants collectively code and apply thematic analysis to qualitative data (29). Rapid qualitative methods are increasingly applied in healthcare research to obtain trustworthy findings through timely and rigorous analysis (33, 34). Rap-GAP was selected because of its efficiency, grounding findings in primary data collection, shared meaning-making among the research team, and prior team experience with this method.

The codes developed in Phase 1 formed the basis for Rap-GAP. Following appropriate training, nine team members (AV, BG, CL, HG, JR, KJW, KR, LP, and YS) coded 1–5 transcripts each, and then five team members (AV, BG, CL, KJW, and LP) and one invited participant (a postdoctoral student with LCB) contributed to group analysis to identify interview themes. The interview participants did not engage in Rap-GAP.

In group analytic sessions, Rap-GAP participants transfer insights from coded transcripts onto a virtual white board where they cluster them into themes that surface and are agreed upon through dialogue. This iterative and interactive process continues until group alignment is reached, leading to reliable data interpretation.

#### Phase 3: second coding for acceptability and appropriateness

In Phase 3, CL and LP second-coded Phase 2 insights, themes, and quotes to assess the acceptability and appropriateness of Patient Voices Video and Stepped Reminders using IOF definitions. To ensure reliability, they cross-checked and met regularly to discuss coding. Discrepancies were occasional, based on the analysts’ retrospective assessment. Those discrepancies centered on whether a participant's comments best illustrated acceptability or appropriateness and were resolved by reviewing Proctor et al.'s construct definitions and reaching analytic agreement. PAB members gave general feedback on the findings.

## Results

Overall, 421 potential participants were invited, and the first 32 respondents who fit the sampling criteria were interviewed. Six interview participants received usual care, 11 received reminders only, nine received video only, and six received both interventions. Recruitment was stopped when those interviews were completed. The measured characteristics of non-respondents (*N* = 409), including those who did not respond to recruitment, declined to participate, or did not attend a scheduled interview, suggest that this population is comparable to that of participants (Supplemental material 3).

Some participants gave reasons for declining participation, and common reasons for declining interviews included a lack of interest in the research study, having competing priorities, and not being comfortable with using MS Teams. The characteristics of the interview participants reflected the underlying trial population. Participants’ characteristics are summarized in Table 2. No new insights emerged during the final one-fifth of all interviews. The identified themes reported here comprehensively captured interview content relevant to intervention acceptability and appropriateness and CFIR-based barriers and facilitators to those outcomes.

**Table 2:** Characteristics of the interview participants.

Participant demographics	*N* (%)
Race	
White	21 (66%)
Black/African American	3 (9%)
Multirace	5 (16%)
Other	3 (9%)
Sex	
Female	15 (47%)
Age at index LDCT	
Mean	64.5
Tobacco status	
Former use	15 (47%)
Current use	17 (53%)
LCS history	
First index scan	17 (53%)
Subsequent scan	15 (47%)
Trial arm	
No reminder; no video	6 (19%)
Reminder; no video	11 (34%)
No reminder; video	9 (28%)
Reminder; video	6 (19%)

The analysis highlighted four key themes: (1) general approval of the Patient Voices video with variation based on LCS experience and informational needs, (2) overwhelming support for Stepped Reminders as helpful and convenient, (3) the Larch Trial interventions were perceived as advantageous and low burden, and (4) preference for timely and multimodal interventions tailored to screening experience and needs. The themes reflect participants’ contemporaneous assessment of acceptability and appropriateness of the interventions as presented during the interview.

Barriers and facilitators within themes are summarized in Table 3 by using the CFIR and clustered into three domains (innovation, individuals, and implementation process) and seven constructs (innovation design, need, motivation, innovation recipients, capability, assessing needs—innovation recipients, and assessing context). We present themes and related subthemes with illustrative quotes lightly edited for clarity and length.

**Table 3:** Barriers and facilitators to the acceptability and appropriateness of the interventions across interview themes.

CFIR domain	CFIR construct	Facilitators	Barriers
Innovation	Innovation design	Patient Voices Video appearance, presentation, and key messages Stepped Reminders design and structure, including outreach and LCS ordering independent of patient visit	Patient Voices Video design features (e.g., language perceived as condescending) and educational content (e.g., perceived as not novel) Frequency of reminders deemed excessive in some cases
Individuals (characteristics subdomain)	Need	Limited screening experience/history	Repeat screening experience Specific knowledge gaps and information need not fulfilled by the interventions (e.g., understanding the cost burden)
	Motivation	Personal belief in and engagement with preventive care Desire to model good health behaviors for loved ones	NR
	Innovation recipients	Individual-level needs (e.g., help remembering to schedule repeat scan) addressed by the interventions	NR
	Capability	NR	Self-perception as being a knowledgeable and proactive patient
Implementation process	Assessing needs—innovation recipients	Implementation of the interventions addressed known patient preferences and needs	Implementation of the interventions was not personalized to all patients
	Assessing context	Implementation of the interventions addressed known barriers to LCS Intervention delivery aligned with routine patient care experience Interventions as developed and implemented support communicating with the PCP	NR

## Theme 1: general approval of the Patient Voices Video with variation based on LCS experience and informational needs

The Patient Voices Video was broadly acceptable, particularly among participants with limited LCS experience or seeking reassurance about screening. The participants highlighted length, tone, pace, and graphics as facilitating engagement by helping viewers to be receptive to information about LCS and encouraging positive emotions:

I think [the video has] a good pace and it's not gonna scare anybody. It'll just provide the information. (ID 122, Male, Current Tobacco Use)

I think for people who have not had a lung scan, I think it is kind of presented in a way that can be easily understood. (ID 152, Female, Current Tobacco Use)

[Video content] is presented well, the graphics are good, calming, nothing in there that would trigger me at all. (ID 340, Male, Current Tobacco Use)

Video appearance succeeded in bolstering key messages. For instance, video images and narration suggesting close connections and support from family and friends were meaningful to the participants who commented on the social aspects of LCS.

I thought it <the video> was good. I like the voice. And information was concise, but you know, concise but important. It made sense and it followed. (ID 122, Male, Current Tobacco Use)

… the video reminded me of that (not being alone), and I think that's an important fact to call out as we shouldn't feel like we're alone in this. (ID 221, Female, Former Tobacco Use)

LCS could be isolating if viewed as an individual experience but sharing it with supportive others could be a source of comfort and strength. By encouraging screening adherence, the video appeared to tap into and affirm participants’ health-related values, including self-care and the importance of modeling good health behaviors for loved ones:

See for me it's also personal. So it's kind of like a personal journey to try to care of my health as I'm getting older and. That video looked like something that was specific to myself. (ID 152, Female, Current Tobacco Use)

Well, the first thing [about the video] is you do [the screening] for your family. You're showing them the way that they need to do [it] to take care of their health. If you show them the right way, you know, then you're being a good mom. (ID 89, Female, Former Tobacco Use)

However, the video was not acceptable to some participants who said it lacked sufficient depth and failed to convey new information. Moreover, the video was judged to be irrelevant when it failed to address specific participant concerns (e.g., how to interpret imaging findings). Participants’ view of themselves as activated and engaged with taking care of their health affected their assessment of the video as being unhelpful or not appropriate to meet their information needs.

Well, like I said, I'm gonna advocate for me. So I wouldn't have needed [the video] but that's good. (ID I290, Male, Current Tobacco Use)

I don't think it would have helped any, only because, umm. I wanna know what my results meant. (ID 276, Male, Current Tobacco Use)

Some participants would have preferred the video to acknowledge possible LCS financial burdens:

They're talking about, you know, it's like having the lung cancer screening is like a mammogram and a colonoscopy again, you're not paying $250 to go have those done, but you're paying $250 for a lung cancer screening. (ID 109, Female, Former Tobacco Use)

Generally, the participants said they would have no problem with receiving the video, whether they believed they needed it or not, suggesting that they found the video generally acceptable, although not always appropriate. The participants also recognized that the additional communication could be helpful and appropriate to others who might forget or be hesitant to screen:

I don't think that I really need [the video], but for somebody [else], I think it's a good thing. Just try to throw it all in, everything in the kitchen sink. Get them to go [to be screened] (ID 89, Female, Former Tobacco Use)

Oh, it wouldn't bother me, but I don't necessarily see—well, maybe it would be a good reminder for people that are distracting or focusing about their health, it might be a good reminder that that's something they should keep in mind. But I don't need that type of prodding. (ID 199, Male, Current Tobacco Use)

### Subtheme 1a: educational content of the video

The participants were open to receiving additional LCS information through the Patient Voices Video, which they viewed as appropriate to address a variety of information needs. For example, the video could reinforce the main messages from the PCP or fill knowledge gaps after SDM conversations:

(The video) helps reinforce—hopefully patients are having these discussions with their doctors, their primary or whoever, but it doesn't hurt. If it was the only way, that's not enough reinforcement. (ID 06, Male, Current Tobacco Use)

I think it's probably good for probably a lot of people. I don't know if their doctors talk to them as thoroughly as my doctor talked to me. I think the video is a good backup plan, right? It ensures that the patient understands what's going on. (ID 123, Female, Former Tobacco Use)

Content about the importance of annual LCS to identify lung cancer early was acceptable and resonated with the participants who liked being reminded why repeat scans are necessary. Their comments highlight a desire for a thorough understanding of LCS that could be satisfied by adherence-focused information boosts independent of the quality and completeness of prior SDM:

I think it's good and I think that it's just it's good reminder of why you do this and you don't do it one time. (ID 45, Male, Former Tobacco Use)

If there is something going on, at least we can catch it early and I understand that's the way that the medical field works. If they find it early, they can help you through it a little better. (ID 290, Male, Current Tobacco Use)

The additional education might counter the disincentivizing effect that consecutive negative results can have on return to screening and be especially relevant for participants who said they were not aware of the need for repeat scans before watching the video:

I suppose even if I wasn't smoking or vaping I would probably still want to get it checked and then if it there was no change, after like a test or two, then I'd probably stop testing. (ID 280, Female, Current Tobacco Use)

Totally new information to me. I've never viewed this as an annual wellness, … preventive care sort of thing, so. It's totally changed my viewpoint on it. (ID 222, Female, Current Tobacco Use)

The video was considered to be potentially appropriate when viewed in the broader context of patient–provider relationships. Reflecting on their overall care experience, the participants identified good communication as central to a positive relationship with their PCP and in that the video could be helpful by encouraging patients to raise LCS with their provider.

Oh yeah, I think in general, I think that someone that uh has never heard of it. I think that might persuade him, at least to have a discussion with the doctor. (ID 45, Male, Former Tobacco Use)

## Theme 2: overwhelming support for stepped reminders as helpful and convenient

The participants expressed great satisfaction with the reminder intervention, describing it as not only acceptable but also appropriate because it was useful and motivating for staying on track with LCS as part of routine care:

I feel pretty good about how they … they keep me informed in a very positive way and on track with what I need to do. (ID 122, Male, Current Tobacco Use)

As best as I can remember, [reminder] worked well and I was definitely satisfied with it and I need it. Without it, I wouldn't have gone, and I do want to go. (ID 340, Male, Current Tobacco Use)

[Receiving a reminder] made me go on my app and find out what screening or whatever I was supposed to do (ID 89, Female, Former Tobacco Use)

Remembering when to talk with the PCP to repeat LCS when it was due can be a barrier to annual screening. A participant noted:

It's not like you can throw [a reminder for next scan] in your calendar, and usually you don't have a year calendar after you get [your LCS scan]. You don't have the next year yet. (ID 89, Female, Former Tobacco Use).

Stepped Reminders addressed this hurdle by offering a helpful nudge toward LCS as an important preventive practice that may be forgotten amid life’s other priorities:

Oh, I think [the reminders are] wonderful. Because life is very busy and … especially the older you get, it has more dumpster fires, things that have to be dealt with now. So this is an immediate crisis. You have to put this first in your priorities and make an appointment for [screening]. (ID 402, Female, Current Tobacco Use)

Stepped Reminders were intentionally designed to fit with KPWA's standard practices, and most participants did perceive the intervention to be similar to other healthcare reminders. This feature facilitated acceptability as the participants commented on their seamless experience across preventive services within the health system:

It's [the Stepped Reminders] about the same because I mean, I get reminders all the time of when I'm supposed to do stuff like I'm due for mammogram soon and colonoscopy. (ID 45, Male, Former Tobacco Use)

I get reminders from [KP] all the time about, they want another blood pressure reading or that it is time to renew a prescription or so forth … I have no problem with that and I'm thankful for that. They stay on top of that, especially medications, they stay on top of it, notifying me. (ID 290, Male, Current Tobacco Use)

### Subtheme 2a: no perceived need to see one’s PCP before scheduling a repeat scan

One of the distinctive features of the Stepped Reminders was that a PCP visit was not required to initiate repeat LCS, thereby reducing demands on provider capacity. The participants strongly agreed that a PCP visit was not necessary before a repeat scan, even though they routinely saw their PCP for other needs:

And [it's good] just knowing that, OK, she's already put in the order for it, you're good to go. Just call and make the appointment. (ID 89, Female, Former Tobacco Use)

I would like to go to my PCP for other checkups or if it's something unusual or I haven't done before, but not something like this, a repeat scan. (ID 92, Male, Former Tobacco Use)

Those who voiced this perspective liked the convenience and cost saving of avoiding a visit, implying that this component of Stepped Reminders was an appropriate fit with their lives and priorities and may help lower barriers to adherence:

I mean the cost of transportation and everything and exposure to people … you don't necessarily need to see these days. (ID 92, Male, Former Tobacco Use)

[Not seeing the doctor] would save me a lot, plus we if there was anything we could talk about at my regular visit. (ID 290, Male, Current Tobacco Use)

## Theme 3: the Larch Trial interventions are perceived as advantageous and low burden

When asked whether there were pros or cons to receiving one, both, or neither of the interventions, the participants perceived the interventions to be acceptable because they imposed such a low burden that even participants who did not need or want them were generally comfortable receiving them, stating that

[Regarding] healthcare I'm not sure that we can be over communicated with, especially if we're not doing it like we should be doing it on a regular basis. (ID 222, Female, Current Tobacco Use).

There were suggestions that video and reminders could be both appropriate if used in tandem to fill knowledge or care gaps and address individual preferences and needs. In that case, viewing the video once could suffice if it conveyed information that the participant already knew, whereas reminders could continue and be enough to strengthen adherence:

I think, respectfully, they're [the interventions] probably both pros. I think there's more pros than cons. You know, it's just a matter of personal choice what people are most comfortable doing, you know? (ID 152, Female, Current Tobacco Use)

I think that the video would be redundant. Maybe sending it one time to encourage somebody to get it. The reminder … that would be much more beneficial to go “Hey, you know you're due in a couple months” and so refer you. (ID 369, Female, Current Tobacco Use)

I think [my doctor] probably would wanna send both. Like I said prior, I don't know all doctors and how they talk to their patients about it [LCS]. I was lucky. I'm pretty sure there's doctors that aren't as thorough. I don't know, but I think you have the whole gamut. And so I think this helped cover for the doctors who don't share as much for the patient. (ID 123, Female, Former Tobacco Use)

An unexpected benefit of the interventions was that they fostered participants’ favorable view of the healthcare system as demonstrating genuine support and concern for patients. This perception enhanced interventions’ acceptability and appropriateness to participants within their care context:

[The video] is cool. It's very encouraging. Seemed like … they really were caring that people should get screened. (ID 369, Female, Current Tobacco Use)

Ah, I think that the fact that you guys see this as a necessity for patients to be aware and kind of take charge of their healthcare in a way, I think it's a wonderful thing because again I would have never thought about getting lung screening until I voiced that I was running [out of breath]. I wasn't coughing or anything, but I wanted to share with my doctor that I may have some concerns about. (ID 158, Female, Former Tobacco Use)

It may also be meaningful and support LCS adherence for participants who said trust in healthcare and providers encouraged their screening:

What keeps me doing [LCS] is I know I need to do it because I trust the people that are telling me I need to do it. (ID 340, Male, Current Tobacco Use)

## Theme 4: preference for timely and multimodal interventions tailored to screening experience and needs

As the results indicate thus far, both interventions tested in the Larch Trial were satisfactory to the participants and addressed identified needs, although not uniformly so, as acceptability and appropriateness could be influenced by factors such as LCS knowledge, LCS experience, and the timing and mode of intervention delivery.

### Subtheme 4a: tailoring interventions by length of experience with LCS

The participants’ comments revealed that the acceptability and appropriateness of the interventions varied by LCS experience. The clearest distinctions emerged when discussing the Patient Voices Video, which was viewed as most appropriate and helpful to inexperienced screeners:

If I'd never had one [LDCT] and my doctors said I needed one, then I think that video would be very helpful beforehand. (ID 109, Female, Former Tobacco Use)

This may be due in part to the emotions that can accompany and sometimes impede screening. Fear, anxiety, and reluctance were not uncommon, even among participants who routinely underwent LCS, as this participant exemplifies:

Well, I'd had one before. You know, I'd always avoided them. I had smoked for 30 something years. I've only not smoked now for three years. So, you know, being a smoker like that, it makes me very nervous to have a lung cancer screening, you know, because you don't want to find out. (ID 109, Female, Former Tobacco Use)

Against this backdrop, the video’s “calming” quality contributed to perceived value to first-time screeners, especially if the video was delivered close to a normal scan (as was done in the trial):

I would think right after (the first screen) and would probably be the best, because that first time around you've probably gotta another level of anxiety about it and the video does seem to be very calming. (ID 221, Female, Former Tobacco Use)

Participants who seemed confident in their own knowledge and ability to adhere to repeat screening found the video less helpful:

Well, maybe for some people, if it was their first, or even their second scan. But I've had three at least, four, and I'm totally convinced that I will get them as soon as I can

… but for rescheduling [LDCT], I think [the video] might be a bit redundant. It seems more introductory. (ID 221, Female, Former Tobacco Use)

Stepped Reminders were also said to be most valuable and appropriate to patients new to LCS, whereas they may be inappropriate for experienced screeners:

I really think once someone becomes aware of these screenings, reminders for the first couple of years are really important. (ID 58, Female, Former Tobacco Use)

The people who have kind of done [LCS] consistently, it's not so necessary and I think for first timers, you know it's probably a good idea. (ID 122, Male, Current Tobacco Use)

### Subtheme 4b: preferred timing of intervention delivery

Preferences on timing of delivering the interventions varied for both Patient Voices Video and Stepped Reminders and influenced appropriateness. As part of the trial, the video link was sent to patients 3 weeks after a scan with no abnormal findings, but the participants disagreed on whether that was the right timing. Some said it made sense to reinforce both feelings of reassurance from a negative scan and the need to return in 12 months. Others maintained that the video should be shown before patients are due to remind them of the reason for repeat scans and when to schedule.

Interviewer: We're sending this video after people get their negative lung scan. Is it the best time to send this or is there another time that you think would be better?

Participant: Before it's time for your next one, just to let them know … things change in a year..[Send the video] a couple months before [next scan], just a little jog [to] your memory. (ID 89, Female, Former Tobacco Use)

No, after a negative scan that would be a good time to show the video because it would remind them that they got peace of mind now, when they wouldn't realize that on their own. (ID 199, Male, Current Tobacco Use)

I think 3 weeks to a month afterwards [after LCS LDCT] it'd be good because it keeps it in their mind and then they realize, “Oh yeah, I gotta go back for one"—kind of plants the seed. (ID 91, Female, Former Tobacco Use)

These comments may indicate a need for additional education close to the repeat LDCT, given that the content of the Patient Voices video was not designed for delivery when due.

When commenting on reminders, the participants said that repeated outreach was acceptable and even needed in some cases. A participant who might have received Stepped Reminders as part of the Larch Trial welcomed an intensive approach:

You're having a lung scan every year, so this year they got in touch with me and they're like, poke, poke, poke. You need to get a lung screening and then I put it off because it was super crazy earlier this year and then they were like poke, poke, poke, you know, so I love Kaiser for that. (ID 280, Female, Current Tobacco Use)

Although multiple reminders were mostly perceived as helpful and appropriate, some participants cautioned that repeated outreach may feel bothersome or aggressive and be largely ineffective.

Just don't keep throwing [reminder] at [the patient], cause I don't think that works. Things that kind of lead towards that but aren't necessarily pointed directly, having the gun pointed directly at you. (ID 340, Male, Current Tobacco Use

Ultimately, the participants agreed that paying attention to one or multiple reminders is a personal preference that varies from patient to patient, underscoring the importance of tailoring strategies to reach intended recipients, while acknowledging their autonomy in making healthcare choices:

Like I said, they can tear up the letter or whatever. I mean, they don't have to. They can shut off their clear the message off your computer, but I don't know. Attempts were made. I think it's better than doing that than doing nothing. (ID 41, Male, Former Tobacco Use)

### Subtheme 4c: preferred method of intervention delivery

As with intervention timing, the participants differed in their preferred method of receiving video and reminders. Within the Larch Trial, the two interventions were predominantly delivered through the patient portal, a method that the participants found broadly acceptable and appropriate because it matched how they usually received notifications and communicated with the health system:

For me, getting just an email saying I've got a new message in my Kaiser [portal]would be fine … might have to download [the video] if there were one. (ID 340, Male, Current Tobacco Use)

You have a new message or you have a new test result. Whatever I know those are coming because it is expected, you know. (ID 144, Male, Current Tobacco Use)

I'm in front of my computer a lot so accessing My Chart is generally the quickest easiest way for me to see communications from my health team. (ID 409, Male, Current Tobacco Use)

Some participants, however, preferred receiving reminders through telephone or mailed letters, while still others said they were comfortable with or needed multiple outreach methods.

… if I get a [voicemail] message from PCP saying, “Hey, just wanted to remind—is calling to remind you that, you know, you got this lung cancer screening coming up, make sure you make your appointment.” I'm probably going to listen to that. (ID 144, Male, Current Tobacco Use)

I love a real phone message, and I love it if it gives you a way to get back. (ID 402, Female, Current Tobacco Use)

And so it comes up again before they send you a letter in the mail. Our department has been trying to call you to to schedule or something or other and you're going and one of the times they do it, you go. My God, this is terrible. They've let me know four times. And you, you actually take it and Scotch tape it to your bathroom mirrors. And then you get it done the 4th day after that. (ID 402, Female, Current Tobacco Use)

## Discussion

Overall, the findings indicate that Larch Trial interventions are generally acceptable and appropriate for LCS implementation to improve patient knowledge and remind patients when they are due for repeat LCS. We tested two patient-centered interventions in the Larch Trial to address identified barriers to adherence to repeat screening. Currently, few effective LCS interventions exist to improve adherence to annual screening, and therefore, identifying key determinants of intervention acceptability and appropriateness is important for futuristic purposes. Although both interventions were generally acceptable and appropriate to the participants, patient factors (LCS experience, knowledge, communication preferences, and attitudes), as well as intervention content, delivery modes, and the underlying healthcare ecosystem, are important context for integration into LCS programs.

Our findings partly align with Sedani et al.'s CFIR-informed systematic review of multilevel factors influencing LCS, while contributing new insights into designing effective interventions to improve adherence (35). As in our study, Sedani et al.’s reported that patients’ knowledge of LCS, desire to care for their health, and high value placed on prevention facilitated screening, However, our interviews revealed that beyond LCS knowledge and proactive health attitudes, length of experience with LCS can also shape patients’ perceptions of repeat screening and relative interventions.

Patients participating in LCS have knowledge gaps regarding their understanding of the purpose of screening and when to return as a result of imaging (10). Although many participants found the video reassuring and informative, others viewed it as redundant or overly simplified, indicating that additional information needs might be directed at to those with limited LCS experience. Patients’ reflections align with other evidence from the trial. Eight weeks after scan completion, we surveyed Larch Trial participants to compare survey responses on LCS knowledge, perceptions of tobacco-related stigma, and self-efficacy among those randomized to health communication vs. those who were not (25). We found that the health communication intervention increased knowledge to return in one year by 13% in the intervention arm vs. control [Relative risk (RR) = 1.13, 95% confidence interval (CI) 1.05–1.22, *p*-value < 0.001], and the increase in knowledge was most evident in those who were first screeners (62.0%–83.9%, RR 1.35, 95% CI 1.16–1.58), while there was no difference in those who had screened at least twice (25). Through the interviews, patients identified additional information needs (e.g., cost of screening). LCS-shared decision-making is often considered the point of entry to screening, given the requirements for reimbursement by the Centers for Medicare and Medicaid Services (CMS). However, our interview findings suggest the need for additional education about LCS to supplement SDM conversations and resources (e.g., emotional and financial counseling or materials) to fully meet patient needs.

We tested a multilevel reminder intervention that initiates repeat LCS with the PCP and includes direct patient outreach to schedule screening. The interview participants were overwhelmingly in favor of continuing screening participation annually without the need for a prior PCP visit, similar to how breast cancer screening is conducted (36). In LCS, approximately 85% of those who screen will have negative or benign findings, prompting repeat screening in 12 months (37). The study interventions were tested only in a population that was due for repeat screening, who might be the most appropriate for receiving standardized LDCT orders (38). Although LCS scans in the trial were prompted by LCS coordination, in healthcare systems with limited resources or capacity, standardized ordering of annual LCS scans could efficiently reduce clinical workload and increase annual adherence. Not surprisingly, patients favored multimodal and tailored communication, as shown in other cancer screening studies (39). Reminder-based interventions to improve adherence to annual screening are likely an essential component of LCS programs (whether centralized or hybrid) (40, 41) to ensure timely return to screening, and reducing barriers to screening caused by unnecessary appointments could increase screening participation.

Any LCS intervention tested and implemented within clinical care needs to reinforce trust between the patient and the PCP. Trusting relationships may be a key mechanism through which implementation outcomes can be achieved (42), especially in LCS where decisions to screen have been linked to patients’ perceptions of person-centered interactions with providers that fostered confidence and mitigated test-related anxiety (43). For delivery of both Larch Trial interventions, patient acceptability and appropriateness were enhanced by attention to the underlying health system communication norms to ensure a seamless patient experience. This finding underscores the extent to which the inherently complex and multilevel nature of healthcare contexts affects individual experience of intervention (44).

The interventions were intentionally designed to be embedded in routine care and leverage multimodal communications in use within the health system. This purposeful and multipronged approach was favored by participants, but it depends largely on system-level factors, such as technology infrastructure and resource allocation, which have been widely shown to be strong determinants of LCS outcomes in US and international settings (45).

Ultimately, the underlying communication system norms within the healthcare system were a strong facilitator for effective delivery of LCS interventions. Many participants were accustomed to using the electronic patient portal to interact with providers virtually and liked receiving additional education and reminders in the same way. Trial data showed that 89% of health communication intervention recipients who were active on the patient portal opened the message with the video link and 47% (*n* = 103) of 363 surveyed health communication recipients reported watching the video (25). The participants noted that the Larch Trial interventions demonstrated that their health system cared enough to go “above and beyond” by testing new ways to care for their health. Beyond improvements in clinical outcomes, this finding suggests that developing innovations that are rooted in patient preferences and needs can build overall trust and increase satisfaction, offering a possible system-level incentive for adoption of interventions. Although systems with limited resources may have less capacity to develop tailored solutions for the populations they serve, these insights can guide efforts to adapt existing interventions, so that they are well received by the patients most likely to benefit from them.

The findings of this study suggest approaches to mitigate knowledge and communication gaps that represent barriers to adherence but do not address other challenges to timely completion of annual LCS. Multiple patient-level factors (e.g., health insurance status, distance from imaging centers, and other logistics) (46, 47), provider-level factors (e.g., familiarity with screening guidelines and limited time during visits) (47, 48), and system-level factors (e.g., lack of LCS prioritization and inadequate resources) (47) are critical to adherence and must be targeted to improve LCS outcomes.

## Study strengths and limitations

The strengths of this study are as follows: A robust sample and comprehensive analysis supported thematic saturation. Grounding data collection and analysis in the CFIR and IOF provided a rigorous foundation for understanding the barriers, facilitators, and outcomes of developing and implementing interventions that meet patients’ needs. Employing the Rap-GAP group analysis method leveraged team members’ diverse backgrounds and perspectives to enhance data interpretation. Thoughtful methodological choices strengthened reliability throughout the analysis.

The limitations are as follows: Patient Voices Video was a part of a broader health communication intervention that included printed messaging with the same key messages to extend the reach of the intervention. However, the video interview questions primarily assessed the novelty of digital communication. Thus, we had limited information about the full intervention.

We interviewed trial participants after their final outcomes were measured for the main trial. This meant that patient acceptability and appropriateness were assessed during a hypothetical situation for the participant, rather than at the time in which interventions were delivered and received. Given this study design choice, patient perspectives might be different from the ones in this study if patient acceptability and appropriateness were assessed at the time of delivery. This context might affect the applicability of our findings, and therefore, assessments should be made when considering intervention adoption and scale-up beyond the Larch Trial. We did not share data or findings with participants for member checking.

Our methods promoted thematic saturation across the study sample, but saturation was not examined by subgroup, and additional data patterns remained unexplored. The study sample was drawn from all trial arms, but the number of participants per arm differed, which might have under- or over-represented some themes.

Last, the study findings might be influenced by the biases and assumptions of a well-educated research team comprising individuals with little or no direct experience of LCS. This limitation was mitigated in part by consultations and input from the PAB throughout this study.

## Conclusion

Overall, LCS interventions designed to address patient barriers were acceptable and appropriate to patients within an embedded pragmatic clinical trial. Intervention development and implementation were shaped by the healthcare organization in which the trial took place, warranting caution in generalizing the findings beyond the context of an integrated delivery system. Nevertheless, this study offers lessons applicable across healthcare organizations seeking to drive patient-centered solutions to LCS adherence gaps. Future LCS trials should consider patient information needs, intervention delivery cadence, and reducing healthcare system access barriers to further improve LCS adherence.

## Data Availability

The original contributions presented in the study are included in the article/Supplementary Material; further inquiries can be directed to the corresponding author.
