# Supplementary material for: I need it. Without it, I wouldn't have gone, and I do want to go: patient perspectives on the acceptability and appropriateness of two patient-centered interventions to improve adherence to annual lung cancer screening
Source: Front Health Serv. 2026 Sep 23;6:1810956. doi: 10.3389/frhs.2026.1810956 (PMC13642276; doi:10.3389/frhs.2026.1810956)
Supplement: Supplementary file 1 [file Table1.docx]

Supplementary material 1

# Patient interview guide – Larch Trial

## Introduction

Hello. [PARTICIPANT NAME]?

Hi, my name is [NAME] and, before we go further, to make sure I am talking to the right person, can I confirm your date of birth?

[Patient confirms DOB]: Great. Thank you.

Thank you for agreeing to talk with me today. We are conducting interviews with Kaiser Permanente Washington members who might be eligible for lung cancer screening to learn about their experience. We are hoping that your input will improve how Kaiser Permanente offers this care for patients like you.

As we mentioned in our written materials, this interview is part of a research study which Kaiser Permanente Washington Providers and Clinics are participating in to improve communication about lung cancer screening for our members. The study is funded by the National Institute of Health.

This interview will take about 60 minutes. Afterwards, we will mail you a $60 in cash to thank you for your time. If you agree to the interview, your answers will be kept private and will never be shared in any way that could be linked back to you.

None of the information from the interview will be put into your medical record or shared with your doctor. This interview will be recorded and transcribed. We will not use your name on the transcript. We may use quotes from the interview in reports, papers, or presentations – but your name or anything that could identify you will be removed.

Participating in this interview is up to you. You are free to say no now or later. Either way, there will be no penalty. Your decision will not affect your health care or benefits in any way. You may skip any questions you do not want to answer.

Did you receive the information sheet we sent you that contains more information about the study? Do you have any questions?

Would you like to participate in this interview?

Yes______	No______

Do I have your permission to record this conversation? The recording will be kept through June 2031 and then deleted.

Yes______	No______ [If say no, continue interview but focus on bolded questions and only take notes

*If yes:* I will start the recoding now

INTERVIEWER STATES: “I am starting the recording now with patient’s permission.”

*Experience with care at KPWA*

To start, I’d like to talk to you about your experience with care at KPWA in general.

1. Do you have a primary care physician (we refer to them as PCP in this interview)?
   a. (if yes) Tell me about your experience and your rapport with your PCP
      i. Do you feel you can trust this provider? What makes you say that?
      ii. How does your provider share information about preventive care (e.g., recommended tests to prevent illness) with you? Do you get this information in any other way? If so, how?
   b. (if no) Can you tell where and how you get care?

*Experience with LDCT screening – general*

Now I would like to talk about lung cancer screening with you. I’m interested to learn about your experience with lung cancer screening at KPWA and the role this screening plays in your care. I’ll start with how you first started screening for lung cancer and then ask about interactions with your care team when you schedule and come in for screening.

1. Tell me about your lung cancer screening experience.
   a. How was lung cancer screening introduced to you?
   b. What made you want/not want to be screened (at any point in the process)?
   c. What concerns did you have about lung cancer screening?
   d. Do you have those same concerns today?
2. Thinking about your experience with lung cancer screening thus far, how would you compare this experience with receiving other services – especially other preventive care services (like blood work, breast cancer screening, colon cancer screening) – at KPWA?
3. What would motivate you to continue lung cancer screening?
4. What (if anything) gets in the way of returning for your next screening scan when you’re due? This could be anything from lack of time, to lack of transportation, feeling the procedure is uncomfortable, or any other factor.
   a. How could your provider or care team help you overcome the challenges you mentioned?
5. What do you think is important for your PCP to know about you when you are thinking about lung cancer screening?

*Introducing the Larch interventions*

As part of the study, we are testing two means of communicating with patient about the importance of lung cancer screening and of scheduling their next screening when due. I’m going to show you a slide that illustrates both means of communication. We will talk about them one at a time.

(Show slide, by sharing screen, focusing on the Patient Voices Video flow only) [SEE ATTACHED SLIDE]

*Experience with Patient Voices video*

Now I’d like to talk to you about what we call the Patient Voices Video. This is a 3-minute video about lung cancer screening that we created and are testing with KPWA members to support repeat screening.

In the past few weeks or months, you might have been invited via secure message or a letter in the mail to view this video. We will watch the video now and then we can discuss it. Now I am going to open up the video and share my screen so we can watch it together.

NOTES:

- IMPORTANT! To share the sound of the video with the interviewee, you must do this **FIRST**:
  1. Click “Share content” on top right of Teams panel. It’s the button with an arrow pointing up. [Do not share yet – just click the button].
  2. Click slider to turn on “Include computer sound.”
  3. THEN, pick the document and share it with the interviewee.
- If participant is not able to view shared screen, verbally give them the video URL – “kplung.org/test” and have them watch it on their own – e.g., on their phone - while you wait and then resume the interview when they have watched it].
- Another way the patient can watch the video on their own is to use QR code below if you copy and paste it into a Word document that you can share.

<https://kplung.org/test>

<https://kplung.org/test>

[Share screen to watch the video.]

[Check that patient can hear recording before continuing] **Can you hear the recording?**

6. Now that you have watched the video, what’s your reaction? If you also viewed the video at an earlier time (i.e., before this interview) feel free to describe your reaction then, too.
7. What did you like or not like about the experience, from accessing the video to the content of the video, or anything else about it?
   a. What appealed to you about the content? Did you see someone like yourself in this video?
   b. What did you not like or did not apply to you?
   c. What did you get out of the video? In other words, what did you understand to be message of this video?
   d. What did you learn that was new or unexpected?
   e. Is there any other information that you think is missing (e.g., what other messages should be conveyed)?
   f. Can you describe how you felt during and after watching the video (e.g., anxious, reassured)?
   g. Is this video a type of communication you’d like to receive from your PCP? Why or why not?
   h. Would you want to talk to your PCP about watching this video and initiate a conversation with them about lung cancer screening? Why or why not?
   i. When do you think is the best time to view a video like this? Right after a screening (when it is a long time before you are due again) or at some other time?
8. Would you recommend the experience of watching the video to a friend or loved one needing to have regular lung cancer screening? Why or why not?
   a. Would you recommend that they ask their provider about returning for annual lung cancer screening? Why or why not?
9. What suggestions do you have to improve the experience of receiving and watching the Patient Voices video? This could be anything from accessing the video, to images and spoken words, to length of the video.
   a. What could be done to make the video accessible to most people, e.g., those hard of hearing, with vision problems, or who speak English as a second language?

*Experience with Stepped Reminders*

We are also testing reminders to help people schedule their lung cancer screening when due – we call this program Stepped Reminders. It’s possible you have received reminders to schedule a lung cancer screening appointment as part of this program we’re testing; if so, I’d love to hear about that, but if you haven’t (or don’t remember) don’t worry! I am going to describe the program to you now. Here’s how it works:

(Show slide again by sharing screen, focusing on the Stepped Reminders flow only)

- Eight weeks before scan is due:
  - First, a Medical Assistant sends order for your lung scan for your PCP to sign
  - Once your PCP signs the order, a Medical Assistant reminds you to call radiology to make your lung scan appointment. First she sends you a message in your electronic medical record (if you use that), then a letter, and then up to three phone calls, if you still haven’t scheduled it.

10. Do you recall being contacted and to come back for lung cancer screening? You might have been contacted via secure message, mail, or phone.
    a. If yes:
       i. Do you recall when you were contacted? How (e.g., secure message, letter, phone call) were you contacted? By whom (e.g., someone from your doctor’s office)?
       ii. Did any of these reminders prompt you to schedule your screening appointment? Please explain.
       iii. How satisfied were you with being reminded to schedule your next lung cancer screening? What would you change/improve about the process?
    b. If no:
       i. What’s your reaction to the Stepped Reminders program I just described?
       ii. What did you find appealing or helpful? What would you change?
11. An important part of the lung cancer screening process is the part where the Medical Assistant contacts your PCP to initiate lung cancer screening with you. Your PCP would have sent an order to Radiology for you to screen. This means that you may not need to see your doctor before your screening. What do you think about that? Do you think that is helpful or unhelpful? Why?
12. Based on your recollection and experience and/or the description Stepped Reminders you just heard, how does receiving reminders for lung cancer screening compare with receiving reminders from KPWA for other tests (e.g., routine blood work, other preventive screening)?
    a. Is it similar, better, or worse?
13. We talked about Patient Voices Video and Stepped Reminders as two possible ways to support you to return for your next scan.
    a. What would be the pros and cons of offering both to patients (i.e., sending a link to the video and reminders)?
    b. From your perspective, what else could be done?
14. Those are all my questions about lung cancer screening. Is there anything t else you would like to share about:
    a. Scheduling and receiving lung cancer screening at KPWA.
    b. The Patient Voices video
    c. Screening reminders

Great, thank you! Before I end this interview, I have a few last questions about you. If you prefer not to answer any of them, just let me know.……...

15. Which of the following race categories best describes how you see yourself? [*If multiple selections, use multi-race]*

- American Indian or Alaska Native
- Asian
- Black or African American
- Native Hawaiian or other Pacific Islander
- White
- Other -- If other, please describe: _____________________
- *Multi-race*
- Prefer not to answer

16. Are you Hispanic/Latino/Latinx?

- Non-Hispanic or Non-Latinx
- Hispanic or Latinx
- Prefer not to answer
- Prefer not to answer

17. What best describes your level of education?

- Less than high school
- High school graduate
- Some college
- Four years of college or above
- Prefer not to answer

This concludes our interview. Thank you for participating! You should receive a letter in the next few weeks containing the $60 in cash to thank you for your time.
