# Supplementary material for: I need it. Without it, I wouldn't have gone, and I do want to go: patient perspectives on the acceptability and appropriateness of two patient-centered interventions to improve adherence to annual lung cancer screening
Source: Front Health Serv. 2026 Sep 23;6:1810956. doi: 10.3389/frhs.2026.1810956 (PMC13642276; doi:10.3389/frhs.2026.1810956)
Supplement: Supplementary file 3 [file Table3.docx]

Supplementary material 3

Table S1. Characteristics of recruitment/interview

non-respondents

| **Non-respondent^*^ demographics** | **N (%)** |
| --- | --- |
| **Race** |  |
| White | 319 (78%) |
| Black/African American | 1 (<1%) |
| Unknown | 20 (5%) |
| Other | 70 (17%) |
| **Sex** |  |
| Female | 200 (49%) |
| **Tobacco Status** |  |
| Former use | 185 (45%) |
| Current use | 224 (55%) |
| ^*^ Non-respondents include individuals who did not respond to recruitment, declined to participate, or did not attend a scheduled interview. | |
