# Supplementary material for: I need it. Without it, I wouldn't have gone, and I do want to go: patient perspectives on the acceptability and appropriateness of two patient-centered interventions to improve adherence to annual lung cancer screening
Source: Front Health Serv. 2026 Sep 23;6:1810956. doi: 10.3389/frhs.2026.1810956 (PMC13642276; doi:10.3389/frhs.2026.1810956)
Supplement: Supplementary file 4 [file Table4.docx]

Supplementary material 4: AI prompts and summary of output

Microsoft 365 Copilot, GPT-5 chat model (source: Microsoft Corporation) was used to aid manuscript language copyediting, literature search, and Table 2 preparation.

*Copyediting*

Prompt: Find synonyms for well-resourced systems and under-resourced systems

Output: [Returns output with synonyms commonly used in academic writing and implementation science; includes recommendations for specific reference to staffing, funding, and infrastructure].

Prompt: Find accurate synonyms for LCS adherence to avoid repetition? Only return what is found in academic literature.

Output: [Returns output that includes alternative expressions – including from this study’s authors - to improve manuscript readability]

Prompt: Add this citation to the reference list [citation]

Output: [Adds citation to the manuscript]

*Literature search*

Prompt: Has any article in implementation science cited trust in health care provider as a factor in screening?

Output: [Returns references in implementation science and relevant literature on screening determinants]

Prompt: Provide review articles on multilevel determinants of lung cancer screening.

Output: [Returns three references: Sedani et al, 2022; Reihani et al, 2021; Belachew et al., 2025.

*Table 2 preparation*

Prompt: [Given input of Excel spreadsheet with characteristics of non-respondents]. Give frequencies by race, sex, tobacco status.

Output: [Returns output of counts and percentages]
